# Effects of Switching From Smoking to Vaping on Clozapine Concentration–Dose Ratios and Plasma Clozapine Concentrations: A Systematic Review of Case Reports

**DOI:** 10.1097/FTD.0000000000001476

**Published:** 2026-05-27

**Authors:** Somtochukwu D. Uche, Deborah Robson, Hannah Walsh, Eve Taylor, Ann McNeill

**Affiliations:** Department of Addictions, Institute of Psychiatry, Psychology and Neuroscience, King's College London, London, United Kingdom.

**Keywords:** clozapine, e-cigarettes, smoking cessation, CYP1A2, tobacco smoking, clozapine monitoring

## Abstract

**Introduction:**

Smoking tobacco cigarettes affects the metabolism of several antipsychotic medications by inducing CYP1A2 enzymes. Clozapine is particularly sensitive to this effect, as CYP1A2 induction increases the plasma clearance of the drug, resulting in lower clozapine plasma concentrations and a reduced clozapine concentration–dose (C/D) ratio than that in nonsmokers. Although the increase in clozapine concentrations and C/D ratios after smoking cessation is well-established, the effects of switching to e-cigarettes (electronic cigarettes or vapes) or initiating vaping without prior smoking is not well-understood. This review aimed to address these gaps.

**Methods:**

The literature was searched for articles on patients taking clozapine who used or had used e-cigarettes, including those who switched from tobacco cigarettes. Eligible publications were reviewed and information on patient demographics, psychiatric history, smoking status, clozapine dose, plasma clozapine concentrations, and C/D ratios before and after vaping were extracted. When not reported in the articles, the C/D ratio was calculated using the available plasma clozapine concentrations and corresponding clozapine dose.

**Results:**

Five articles were identified, all case reports describing 5 patients (4 male) who switched from tobacco cigarettes to e-cigarettes while taking clozapine. Switching resulted in increased C/D ratios and plasma clozapine concentrations, although the magnitude of this increase varied among individuals. The most commonly reported symptoms after switching from smoking to vaping are constipation, fatigue, and visual changes.

**Conclusions:**

Based on the small number of case reports, complete switching from tobacco smoking to e-cigarette vaping may have a similar effect on clozapine plasma concentrations and C/D ratios as smoking cessation without vaping. The authors found no research on the impact of vaping on clozapine concentrations and C/D ratios in people who had never smoked.

## Abstract 2

Supplemental Digital Content is Available in the Text.

## BACKGROUND

Tobacco smoking is a leading cause of preventable death and disability worldwide.^1,2^ In most countries, smoking prevalence is notably higher among individuals with mental health conditions than that observed among those without such conditions. In England, smoking prevalence in adults was estimated to be 10.6% in 2024.^3^ Among those with long-term mental health conditions, the proportion of those who smoked tobacco cigarettes was more than double at 24% between 2024 and 2025^4^ and can reach 70% in both patients with schizophrenia^5,6^ and hospitalized mental health patients.^7^ However, people with mental health conditions are just as motivated to stop smoking as are those without.^8,9^

In England, the use of e-cigarettes is also common among people with mental health conditions.^9–11^ Nicotine-containing e-cigarettes (also known as electronic cigarettes, electronic nicotine delivery devices, or nicotine vapes) are widely used smoking cessation aids. Evidence from clinical trials,^12^ surveys,^13^ and population studies^14^ among people with and without mental health conditions indicates that they can be effective in helping people stop smoking.

E-cigarettes do not contain tobacco and instead heat an e-liquid that typically contains nicotine, flavorings, and a base of vegetable glycerin, and/or propylene glycol.^15^ As they are noncombustible, they do not produce smoke and the 80 known carcinogens that are released from tobacco cigarettes.^16^

Heating and inhaling e-liquid constituents may lead to increased harm,^17^ and concerns have been raised regarding flavorings. A recent systematic review indicated that some flavors (eg, cinnamon) may be harmful, but the evidence was predominantly from in vitro studies and chemical analyses.^18^ Overall, e-cigarettes are likely to carry risks to health, but biomarker studies indicate they are less harmful nicotine delivery devices than tobacco cigarettes.^19,20^

Recommendations for e-cigarette use for tobacco smoking cessation vary across national health bodies.^21^ In England, guidance from the National Institute for Health and Care Excellence (NICE) includes nicotine-containing e-cigarettes as a first-line option, on par with combination nicotine replacement therapy, varenicline and cytisine, and behavioral support.^22^ By contrast, organizations in other countries continue to prioritize licensed pharmacotherapies and behavioral intervention.^23,24^

Since 2013, the United Kingdom guidelines have recommended health care to be delivered in smoke-free settings, for smoking cessation support to be offered to those receiving community and inpatient mental health care, and for those unable or unwilling to stop being provided with treatment to temporarily abstain from smoking.^22^ In addition, under the NHS Long-Term Plan in England,^25^ NHS-funded tobacco-dependence treatment is embedded across 3 pathways: acute inpatient care, maternity, and mental health inpatient services. This includes the option to switch to e-cigarettes in inpatient settings for long-term users of specialist mental health and learning disability services. Vaping is now widely integrated into mental health services in England, as almost all NHS mental health trusts permit vaping outdoors, approximately half allow it indoors in designated areas, and a substantial proportion provide e-cigarettes as part of the NHS tobacco dependence treatment pathways.^26^

Against the backdrop of high smoking prevalence, the widespread use of e-cigarettes, and their integration into mental health services, understanding the clinical implications of switching from smoking to vaping is increasingly important. This is particularly critical for people prescribed clozapine, an antipsychotic licensed to treat refractory schizophrenia, as well as other similar antipsychotic drugs such as olanzapine.^27^ Current guidance on smoking while taking clozapine^28–30^ recommends reducing the clozapine dose when the patient stops smoking. Tobacco smoke contains polycyclic aromatic hydrocarbons (PAHs) that induce certain hepatic enzymes, particularly CYP1A2, which metabolizes clozapine and norclozapine.^28^ Because e-cigarettes do not involve combustion, they do not produce PAHs and are, therefore, unlikely to affect CYP1A2 activity. However, this presents a potential risk for patients who switch from smoking to vaping, as they may develop clozapine toxicity when taking a dose that was previously tolerated when CYP1A2 activity is induced by tobacco smoke.

Recent studies have highlighted the need to evaluate how the use of e-cigarettes in general affects clozapine metabolism.^31^ A recent literature review^32^ concluded that “vaping may or may not alter the plasma concentration of clozapine depending on the content.” For people switching from smoking to vaping, it indicated that any effect would primarily occur through CYP1A2 deinduction.

Therefore, a systematic review was conducted to ascertain the impact of vaping in patients treated with clozapine on (1) plasma clozapine concentrations, (2) clozapine concentration–dose (C/D) ratios, (3) blood cell measurements, and (4) psychiatric symptoms. We also sought to elucidate a possible mechanism for this effect.

## METHODS

### Protocol and Registration

This systematic review followed the Preferred Reporting Items for Systematic Reviews and Meta-Analyses (PRISMA) guidelines^33^ and was registered in the PROSPERO online database (PROSPERO Registration: CRD42023453105).

### Search Strategy

A search strategy was developed combining terms relating to e-cigarettes and clozapine, including key words such as “clozapine,” “vaping,” “anti-psychotic,” and “e-cigarettes” (and their word variations). The following databases were searched to locate studies related to clozapine participants who used e-cigarettes:

Cochrane, PubMed, APA Psych Info, Web of Science Core Collection, CINAHL, MEDLINE, and EMBASE (through OVID) (see **Supplementary 1** details the search strategy, **Supplemental Digital Content 1**, http://links.lww.com/TDM/A945). We also searched the reference lists of the included studies for relevant articles.

### Eligibility Criteria

#### Population

Individuals who used clozapine and vaped (either switched between vaping and smoking tobacco cigarettes or recently started vaping without smoking cigarettes).

Studies meeting the following criteria were eligible for inclusion in this review:

1. Study participants exposed to clozapine.
2. Study participants who were either currently using or used e-cigarettes.
3. Study participants older than 18 years.

Studies were excluded if they:

1. Reported participants younger than 18 years
2. Reported e-cigarette or vaping use–associated lung injury.
3. Reported duplicated or overlapping data.
4. Studies on clozapine and e-cigarette exposure in nonhuman species.
5. Participants who reported using e-cigarettes containing illicit drugs (eg, cannabis) or other substances not usually present in (nicotine) vapes.

#### Intervention(s) or Exposure(s)

Exposures included: Clozapine, Vapes/E-cigarettes of any type and their constituents (eg, nicotine, flavorings).

#### Comparator(s) or Control(s)

Participants who used clozapine and either smoked tobacco cigarettes, used both tobacco cigarettes and e-cigarettes (“dual users”), or were neither smoking nor vaping (“nonusers”). We also included studies without comparator interventions.

#### Main Outcomes

Effect of Vaping on Clozapine C/D Ratios and Plasma Concentrations

Additional Outcomes

1. Effect of vaping on blood cell measurements (such as white blood cell count)
2. Possible mechanism by which vaping affects clozapine concentrations.
3. Changes in psychiatric symptoms due to switching from smoking to vaping while using clozapine

#### Study Selection

The search was first performed in August 2023 and repeated on April 29, 2025. After the elimination of duplicates, titles and abstracts were screened [S.U.], and abstracts with insufficient information were retained for full-text appraisal. Full-text screening was conducted by 2 independent reviewers [S.U. and A.M.] against the eligibility criteria preset in our protocol [Prospero ID: CRD42023453105], and any disagreements were settled by discussion with DR. References of relevant articles were screened for additional reports [S.U.]. We used Google Translate for 2 full-text reports written in Dutch, and the translations were reviewed by 3 colleagues from the Netherlands [M.K., J.V., and B.B.].

#### Quality of the Studies

SU and AM appraised the included reports using the JBI critical appraisal tool for case reports.^34^ Reports that fulfilled 7 or more of the 8 questions were deemed to be of high quality, between 4 and 6 moderate quality, whereas those fulfilling 3 or fewer were of low quality (see **Supplementary file 2**, **Supplemental Digital Content 2**, http://links.lww.com/TDM/A946). We also used the CARE checklist to evaluate the completeness and transparency of the case reports (see **Supplementary file 3**, **Supplemental Digital Content 3**, http://links.lww.com/TDM/A947).^35^

#### Data Extraction

SU and AM independently extracted data from the articles using a data extraction sheet. The extracted data included study characteristics, patient demographics, medical and psychiatric history/diagnosis, medications including clozapine dose/route of administration, hospitalization, presenting features before and after switching, plasma clozapine concentrations (and available plasma norclozapine concentrations) before and after switching, smoking and vaping frequency, timeline of switching, and onset of symptoms. For each report, we calculated the C/D ratios (when not given) to ascertain the clozapine clearance rate after switching to vaping. Finally, we assessed the percentage change in clozapine concentrations and clozapine C/D ratios after switching from smoking to vaping.

#### Differences From Protocol

In addition to the psychiatric symptoms detailed in the case reports, we extracted information on any adverse or side effects reported after the initiation of vaping. These terms are sometimes used interchangeably. We were also interested in the side effects and adverse effects of clozapine dose changes.

In some of the case reports, the prescribed clozapine dose when smoking was different from the patient's dose when they started vaping (unshaded rows in Table 1). Therefore, we used C/D ratios, rather than clozapine plasma concentrations, which enabled us to compare clearance rates across the included case reports regardless of the dose changes. Although not a primary outcome, only 2 case reports detailed norclozapine concentrations (Table 1). Hence, we did not include the percentage changes in norclozapine concentrations in our analysis.

**TABLE 1.** Patient Demographics, Clozapine Plasma Concentrations, and C/D Ratios While Smoking and After Switching to E-Cigarettes

First Author, Year, Country	Sex, Age, Race	Psychiatric Diagnosis (Presenting Complaint, Setting)	Smoking				Vaping				% Change in Clozapine Concentrations After Switching to Vaping	% Change in C/D Ratio After Switching to Vaping (Difference)
			Cigarettes Per Day^a^	Clozapine Dose (mg/d)	Clozapine(C) and Norclozapine (NorC)^b^ Concentrations (ng/mL)	C/D^c^ Ratio	Vaping (Approx. Duration of Vaping Before Plasma Concentrations Were Obtained)	Clozapine Dose (mg/d)	Clozapine(C) and Norclozapine (NorC)^b^ Concentrations (ng/mL)	C/D^c^ Ratio		
Nonner, 2016,^36^ Netherlands	M, 45, NR^d^	Schizophrenia paranoid type (none, annual checkup)	NR	400	C = 186^e^	186/400 = 0.47	Y (6 mo)	400	C = 696^e^	696/400 = 1.74	274% increase	270% (1.27)
Berm, 2018,^37^ Netherlands	M, 34, NR	Schizophrenia paranoid type (hallucinations, psychiatry outpatient clinic)	10–15	200^f^	C = 347^g^	347/200 = 1.74	Y (1–2 wks)	100	C = 376^g^	376/100 = 3.76	8% increase	116% (2.02)
Khorassani, 2018,^38^ US	M, 52, white	Schizoaffective disorder Bipolar type (worsening paranoia and feeling anxious, admitted to hospital)	10	550	C = 970^e,h^ NorC = 217	970/550 = 1.76	Y (8 mo)	550	C = 1580 NorC = 260	1580/550 = 2.87	63% increase	63% (1.11)
Kocar, 2018,^39^ Germany	F, 23, white	Paranoid schizophrenia (bizarre delusions and auditory hallucinations, admitted to clinic)	10–15	300	C = ∼350	350/300 = 1.17	Y (2–3 wks)	550	C = 1290	1290/550 = 2.35	269% increase	101% (1.18)
Montville, 2021,^40^ US	M, 28, NR	Schizophrenia (none, outpatient appointments with psychiatrist)	20	400^i^	C = 406^g,j^ NorC = NR	406/400 = 1.02	Y (2–3 mo)	400	C = 809^k^ NorC = 555^L^	809/400 = 2.02	99% increase	98% (1)

Key: Shaded rows represent patients who maintained the same clozapine dose while smoking and vaping; a = Cigarettes per day; b = only if present in report; c = Concentration–Dose Ratio; d = Not Reported (NR); e = trough concentrations; f = Also taking paliperidone 150 mg monthly during this period. Paliperidone is not affected by smoking^41^; g = steady state clozapine concentrations; h = last drawn steady state trough concentrations taken year before admission and before quitting smoking; i = also taking mirtazapine 45 mg Once at Night and Extended-Release venlafaxine 225 mg Once Daily throughout; j = mean of concentrations taken 1 yr (446 ng/mL) and 2 yrs (366 ng/mL) before baseline when on 400-mg clozapine (suppl T1); k = mean of concentrations taken just before second visit (801 ng/mL) and the one 4 weeks after that visit (817 ng/mL); l = mean of concentrations taken just before second visit (490 ng/mL) and the one 4 weeks after that visit (619 ng/mL).

## RESULTS

### Study Characteristics and Patient Demographics

Figure 1 shows the PRISMA flow diagram. We identified 1156 references. After removing 400 duplicate records, 749 records were excluded after title and abstract screening, leaving 7 articles for full-text review (all case reports). None of the studies assessed the effect of vaping on white blood cells within the context of clozapine use or the impact of vaping on clozapine concentrations in people who had no prior experience of smoking.

**FIGURE 1.**
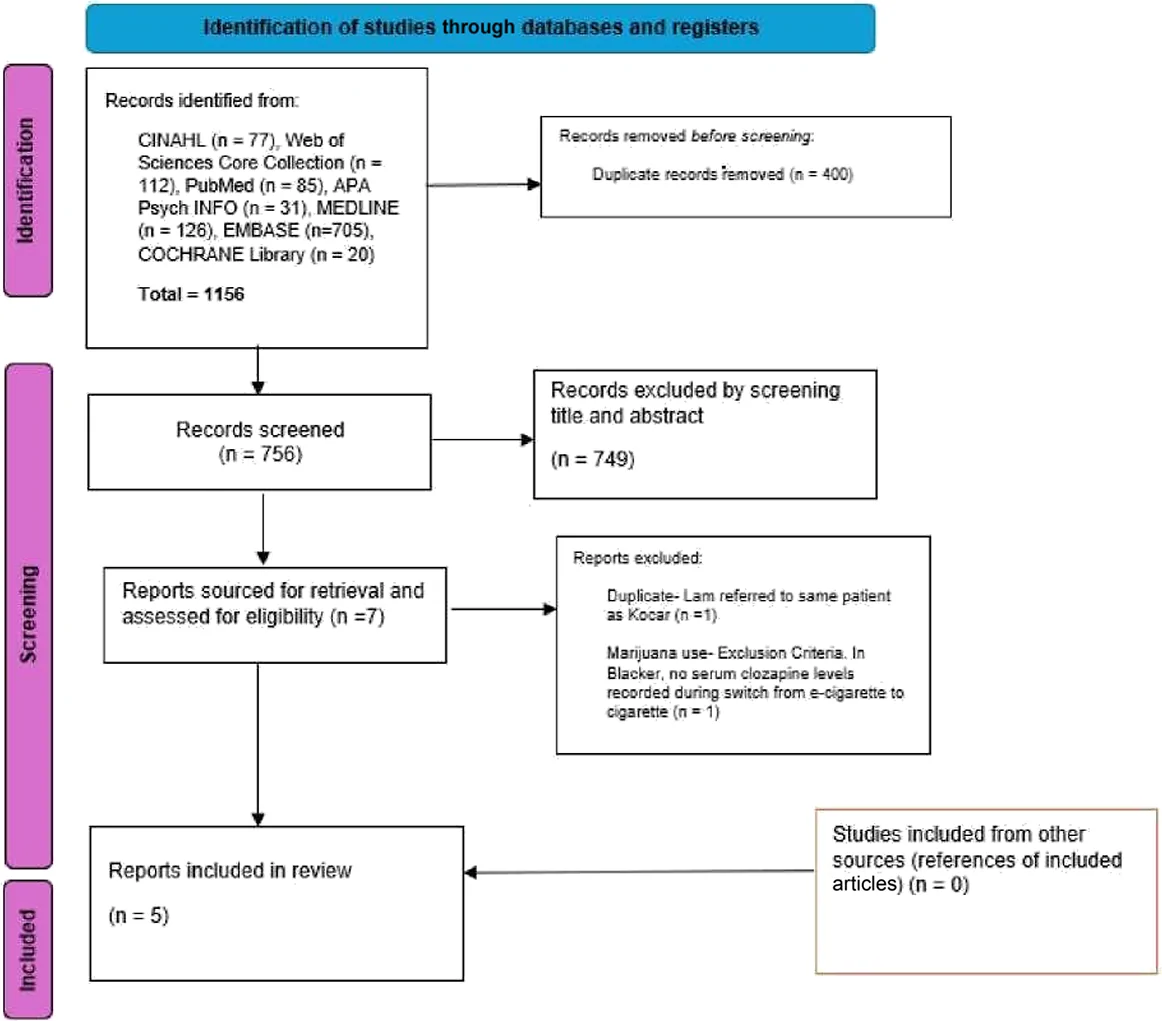
Preferred reporting items for systematic reviews and meta-analyses (PRISMA) diagram/flowchart indicating the articles included in this study.

Two articles were excluded from analysis. A case report by Blacker^42^ was excluded because only one plasma clozapine concentrations was measured when the patient was receiving nicotine replacement therapy. Lam^43^ was excluded, as they described the same patient as Kocar et al^39^ and published their article and a summary of the findings 3 months later. Kocar et al provided context for the results and included a graph providing further information on clozapine plasma concentrations during smoking and the switch from smoking to vaping. Overall, 5 articles^36–40^ published between 2015 and 2021 met our inclusion criteria. Two were from the United States, 2 from the Netherlands, and one from Germany (Table 1).

The case reports included 5 patients, 4 of whom were male, and their mean (SD) age was 36.4 (SD = 10.7) years and ranged between 23 and 52 years. Four patients (3 male and one female) were diagnosed with schizophrenia,^36,37,39,40^ and one (male) was diagnosed with bipolar schizoaffective disorder.^38^ One patient had comorbid somatic condition.^38^

In most reports,^37–40^ the features of tobacco and e-cigarettes (including brand name, strength, and frequency of use) were poorly reported, with only one report^36^ including details of the e-cigarette (Dekang, 18-mg nicotine, tobacco flavor) and none including information on the frequency of use of e-cigarettes. All patients were prescribed oral clozapine, with doses ranging from 200 to 550 mg/d while smoking cigarettes and 100–550 mg/d during or after switching to vaping. Each patient had at least one clozapine plasma concentration recorded before and after switching from smoking to vaping. Table 1 summarizes the characteristics of the cases, clozapine doses, and clozapine concentrations during smoking or vaping.

### The Effect of Vaping on C/D Ratios of Clozapine

All patients included in this review smoked tobacco cigarettes before switching to vaping, although only 3 patients^36,38,40^ maintained their smoking dose, that is, they did not report any change in their prescribed clozapine dose during the switch to vaping (shaded rows in Table 1).

When patients smoked, their C/D ratios ranged from 0.47 to 1.76, compared with 1.74‒3.76 when vaping. Switching from smoking to vaping resulted in an increase in the C/D ratios of clozapine in all 5 cases, with increases ranging from 63%^38^ to 270%^36^ (Table 1). This suggests higher clozapine clearance^44^ during smoking, possibly due to CYP1A2 induction.

### Effect of Vaping on Plasma Clozapine Concentrations

Switching from smoking to vaping resulted in an increase in clozapine concentrations in all cases, including in patients whose clozapine dose was halved during the vaping phase (8% increase in clozapine plasma concentrations).^37^ The increase ranged from 8%^37^ to 274%.^36^

### Patient Presentation and Symptoms (Adverse or Side Effects)

None of the patients reported symptoms related to clozapine toxicity as the main complaint (Table 1). Patients were likely to present during a scheduled outpatient appointment or with worsening psychiatric symptoms, which led to a series of investigations that identified abnormalities in the plasma concentrations of clozapine (Tables 1 and 2).

**TABLE 2.** Symptoms Associated With Raised Clozapine Concentrations, Clinical Interventions After Raised Concentrations Were Discovered and Post-treatment Symptoms

Author, Year, Country	Clozapine Dose While Vaping (mg/d)	Clozapine (C) and Norclozapine (NorC)^a^ Concentrations (ng/mL) While Vaping	Symptoms Associated With Raised Clozapine Concentrations	Clinical Intervention	Smoking/Vaping Status After Intervention	Clozapine (C) and Norclozapine (NorC)^a^ Concentrations (ng/mL) Postclinical Intervention	Symptoms After Intervention
Nonner, 2016,^36^ Netherlands	400	C = 696^b^	Increased fatigue, salivation, involuntary muscle contractions in face, constipation, visual deterioration, tremors, difficulty emptying bladder	Dose reduced gradually to 300 mg/d	Not reported	C = 551	Saliva flow greatly reduced. Other symptoms no longer present
Berm, 2018,^37^ Netherlands	100	C = 376^c^	Drowsiness, difficulty getting out of bed	Dose unchanged as symptoms deemed to not be related to clozapine concentration but rather negative symptom	Dual use: Smoking 5 or more traditional cigarettes and using e-cigarettes	C = 91	Unchanged (possible negative symptom)
Khorassani, 2018,^38^ US	550	C = 1580 NorC = 260	Hypersomnia, amotivation, anergia, low concentration (focus)	Dose reduced to 500 mg/d	Dual use: Likely smoking and e-cigarettes	C = 889 NorC = 219	Not reported
Kocar 2018,^39^ Germany	550	C = 1290	Binge eating, blurry vision, drowsiness	Dose reduced gradually to 250 mg/d	Continues e-cigarettes alone	C = ∼525	Symptoms of clozapine overdosing faded
Montville, 2021,^40^ US	400	C = 809^c^ NorC = 555^d^	No psychiatric concerns New-onset constipation	Dose unchanged as patient voiced desire to return to smoking cigarettes	Dual use: Half a pack of cigarettes alternating with the use of an e-cigarette	C = 633 NorC = 459	2 wks after clozapine concentrations of 633 ng/mL were recorded, patient developed worsening psychotic symptoms, and around this time they started to exclusively smoke 1 pack/d, and self-initiated haloperidol 5 mg/d 1 mo later patient reported symptom improvement and constipation resolved

Key: Shaded rows represent patients who maintained the same clozapine dose while smoking and vaping; a = only if present in report; b = trough concentrations; c = steady state clozapine concentrations; d = mean of concentrations taken just before second visit (490 ng/mL) and the one 4 weeks after that visit (619 ng/mL).

Four patients^36,38–40^ reported adverse physical symptoms after switching to vaping. The most commonly reported symptoms were constipation (n = 2), increased fatigue or anergia (n = 2), and visual changes (n = 2). These were largely not volunteered on presentation but were described in retrospect as the consultation progressed. Three of these patients^36,39,40^ reported that their clozapine-related symptoms subsided once clozapine plasma concentrations decreased, either by returning to smoking or by a decrease in clozapine dose (Table 2). The remaining patient, whose dose was decreased by 50 mg after raised concentrations were found,^38^ described 4 symptoms that potentially led them to quit their job; this patient also reported worsening psychosis and was admitted to the hospital.

The patient who did not report adverse physical symptoms had the lowest percentage increase in plasma clozapine concentrations (8%).^37^ This patient's clozapine dose was halved while vaping and they reported drowsiness and hallucinations, but these were deemed symptoms of active psychosis rather than clozapine toxicity.

### Quality Assessment

As per the CARE checklist,^35^ none of the reports included the term “case report” in the title or keywords. Some reports lacked a clear statement indicating that informed consent was obtained, except for the case reports presented by Kocar (written informed consent)^39^ and Khorassani (verbal consent).^38^

Using the JBI Critical Appraisal Checklist for Case Reports,^34^ 3 of the reports^36,37,40^ were rated as high quality, as they detailed a clear description of the patient's demographics, presenting complaints, clinical conditions, and interventions. The other 2 reports^38,39^ were letters to the editors with case reports/presentations and were classified as moderate quality, scoring positively in all domains apart from the description of the diagnostic methods, results, and treatment/intervention. Owing to the paucity of reports, we excluded none based on quality.

## DISCUSSION

This systematic review identified 5 case reports describing clozapine plasma concentrations in patients who switched from smoking tobacco cigarettes to e-cigarette vaping. Three of these patients were initially administered the same clozapine dose while smoking and vaping, whereas for the other 2, the clozapine dose either increased^39^ or decreased^37^ around the time of switching to vaping. We calculated the clozapine C/D ratios to enable comparisons of plasma concentrations during smoking and vaping across 5 reports. In all cases, the C/D ratios and plasma clozapine concentrations increased, but the extent of increase differed widely. Four of the 5 patients experienced possible adverse effects of clozapine after switching to e-cigarettes.

Among the 5 patients, clozapine C/D ratios increased by 63%–270%, with a mean increase in the C/D ratio of approximately 130%. There is a paucity of published guidelines from major clinical bodies regarding dose-related reference ranges for both C/D ratios and plasma clozapine concentrations. However, based on a meta-analysis, published ranges for clozapine C/D ratios^45^ among smokers typically are around 0.6–1.4 ng/mL per mg/d, whereas in nonsmokers, they are approximately 1.2–2.4, indicating an increased clozapine clearance on smoking. In our cases, smoking (pre-vaping) C/D ratios were within or close to the smoking range, whereas after switching from smoking to vaping, the ratios shifted to the nonsmoking range or above. Therefore, the observed increases aligned with the expected transition from a smoking to a nonsmoking metabolic state after the removal of cigarette smoke.

The increase in plasma clozapine concentrations observed on switching to vaping in our case reports is also comparable to the effects of completely stopping smoking, as seen in a study where the mean clozapine plasma concentrations among 9 patients almost doubled after smoking cessation.^46^ Similar increases in clozapine concentrations have been noted in Asian subpopulations^47^ and in inpatient settings where smoking is banned.^48^

The reason for the variability in the C/D ratio and plasma clozapine concentrations increase is unclear but not unique to our sample. Data from a large therapeutic drug monitoring audit showed that the median plasma clozapine concentrations were approximately 40%–60% higher in nonsmokers than that in smokers, with the greatest differences observed at doses of 151–250 mg/d (approximately 59% in women and 76% in men).^49^ Inter- and intrapersonal differences (eg, treatment adherence, age, race, genetic variation in the CYP 1A2 phenotype, pre-existing kidney disease, and concurrent medications) may have contributed to the variability observed.^44,50^

Heightened CYP1A2 activity in smokers is caused by PAHs produced from the incomplete burning of tobacco. However, nicotine has no effect on CYP1A2 activity.^51^ Overall, our findings supported the hypothesis that vaping halts or reduces CYP1A2 induction, an effect comparable with that of smoking cessation.

Two patients^37,39^ who switched to e-cigarettes while their clozapine dose was altered had their raised clozapine plasma concentrations picked up more quickly (within 3 weeks) than those who did not (for whom the maximum period was 8 months), potentially due to more frequent follow-up appointments. Although none of the cases reported fatal adverse effects, patients on clozapine can develop clinically significant side effects, such as myoclonus or seizures, after smoking cessation, likely due to high plasma concentrations.^46,47^

Patients^36,38–40^ who reported adverse effects of elevated clozapine concentrations recorded a higher percentage increase in plasma clozapine than the patient^37^ who did not report physical effects. These findings are consistent with those of previous studies showing that high clozapine plasma concentrations are associated with an increased risk of concentration-related side effects such as sedation and seizures.^28,46,52^

Worsening psychotic symptoms may be expected should one take up smoking cigarettes due to CYP1A2 induction and reduced clozapine plasma concentrations^53,54^; however, in one patient,^38^ worsening psychosis occurred in the presence of raised clozapine concentrations. Notably, none of the other patients in the reports experienced a worsening of psychotic symptoms after switching to vaping.

These reports suggest that some patients (and clinicians) may not understand the effects of vaping on the pharmacological profile of their medication or that smoking tobacco cigarettes is distinct from vaping. If patients do not perceive vaping to be significantly different from smoking or underestimate its impact on clozapine metabolism, they are unlikely to report this change to their clinical team. Considering the potential effects of smoking cessation and vaping on clozapine C/D ratios and plasma clozapine concentrations, routinely checking the smoking and vaping status at each contact will support the timely recognition of changes in clozapine concentrations or symptoms.

### Limitations and Strengths

Although we ran our search using 7 databases, we were only able to identify 5 articles and all case reports. Therefore, we were unable to perform a quantitative synthesis. Details about smoking and vaping behaviors, including the duration of smoking and/or vaping before clozapine concentrations were collected, were all subject to bias, as is the nature of case reports. While we scored 3 reports as high quality,^36,37,40^ 2 were moderate,^38,39^ which were included due to the scarcity of evidence on the topic. All case reports indicated that the patients switched from smoking to vaping. However, none of the reports contained information on vaping frequency, and only one case report provided details on the type of e-cigarette used.^36^ This could be useful in future analyses because vaping frequency and device type can affect the physiological profile of nicotine.^55^ Because only one case report detailed the type of e-cigarette used, we were unable to comment on the effect, if any, of various vaping devices on clozapine concentrations.

Some case reports did not contain information on how long after dose administration blood was drawn. This may have affected the comparability of smoking and vaping C/D ratios across reports. Future research, including case reports, should specify when the sample used to determine the clozapine plasma concentrations was collected, as clozapine plasma concentrations are a measure of clearance and can vary depending on the time from the dose. Using “trough concentrations” may help improve comparability across reports and studies.

In addition, all the patients included in the study smoked before vaping. Hence, reports and studies including people who did not smoke and have only taken up vaping would also make a useful contribution to the literature. Among these reports, there was no information on the effect of vaping in patients taking clozapine and its effect on blood cell count. Nevertheless, our review is the first systematic review and the most current collation and analysis of clozapine concentrations after switching from smoking to vaping.

### Clinical Implications

None of the patients in the case reports mentioned a switch to vaping or symptoms of clozapine toxicity as their main presenting complaint, and only revealed both later in the consultation. Our findings emphasize the importance of a thorough smoking and e-cigarette history in psychiatry, as well as the acute clinical environment where patients may present, to avoid missing any adverse effects. The latest prescription guidelines on clozapine in the United Kingdom^28^ recommend that clinicians gradually reduce clozapine doses by 25% over the first week after the patient has stopped smoking. Clinical presentation, adverse effects, and plasma concentrations should be used to tailor the dosage, and plasma concentrations should be checked one week after smoking cessation. During this period, patients should be monitored for signs of clozapine toxicity, particularly in the first few weeks after smoking cessation or switching to vaping. These include signs and symptoms such as sedation, hypersalivation, constipation, tachycardia, seizures, or confusion. If a patient continues to smoke while vaping, physicians should continue to manage the patient as a smoker until abstinence is confirmed because tobacco smoke will still have an inductive effect on clozapine metabolism. Monitoring cigarette smoking frequency and clozapine plasma concentrations is imperative during this time, as the concentrations of CYP1A2 induction and plasma clozapine clearance could vary based on the frequency of cigarettes smoked per day.^48^ Overall, patients should be encouraged to stop smoking, and doses should be titrated based on plasma concentrations until smoking cessation is achieved.

## CONCLUSIONS

This systematic review measured the effect of switching from tobacco to electronic cigarettes on plasma clozapine concentrations and concentration–dose (C/D) ratios in 5 case reports. Overall, our findings from a synthesis of these 5 reports suggest that switching completely to vaping may have an effect similar to that observed when people stop smoking and do not vape, by raising clozapine plasma concentrations and C/D ratios. Therefore, someone who has switched entirely to vaping can be identified as a nonsmoker in relation to clozapine monitoring, although regular monitoring will be necessary in case of smoking relapse.

## Supplementary Material

## References

[1] Brauer M Roth GA Aravkin AY . Global burden and strength of evidence for 88 risk factors in 204 countries and 811 subnational locations, 1990–2021: a systematic analysis for the Global Burden of Disease Study 2021. Lancet. 2024;403:2162–2203. 10.1016/S0140-6736(24)00933-4 PMC11120204 38762324

[2] World Health Organization. Tobacco. World Health Organization; 2023. Available at: https://www.who.int/newsroom/fact-sheets/detail/tobacco. Accessed December 10, 2024.

[3] Office for National Statistics. Adult smoking habits in the UK: 2024. Adult Smoking Habits in the UK—Office for National Statistics; 2025. Accessed November 21, 2025.

[4] Office for Health Improvement and Disparities. Public Health Profiles. 2025. Available at: https://fingertips.phe.org.uk/. Accessed November 21, 2025.

[5] Wu C Dagg P Molgat C . Smoking prevalence and correlates among inpatients with schizophrenia or schizoaffective disorder. Sci Rep. 2025;15:18508. 10.1038/s41598-025-93256-2 PMC12117101 40425774

[6] Ziaaddini H Kheradmand A Vahabi M. Prevalence of cigarette smoking in schizophrenic patients compared to other hospital admitted psychiatric patients. Addict Health. 2009;1:38–42. PMC3905500 24494081

[7] Royal College of Physicians, Royal College of Psychiatrists. Smoking and mental health; 2013. Available at: https://www.rcp.ac.uk/media/2yudsitb/smoking_and_mental_health_-_full_report_web_0_0.pdf. Accessed January 1, 2025.

[8] Yimsaard P Gravely S Meng G . Differences in smoking cessation behaviors and vaping status among adult daily smokers with and without depression, anxiety, and alcohol use: findings from the 2018 and 2020 international tobacco control four country smoking and vaping (ITC 4CV) surveys. Int J Ment Health Addict. 2023;22:3433–3450. 10.1007/s11469-023-01058-2 PMC11670891 39735821

[9] Brose LS Brown J Robson D . Mental health, smoking, harm reduction and quit attempts—a population survey in England. BMC Public Health. 2020;20:1237. 10.1186/s12889-020-09308-x PMC7427923 32795286

[10] Do EK Aarvig K Panigrahi G . Past-year mental illness diagnosis and E-cigarette use status among a college-aged sample: findings from the National college Health Assessment (2017-2019). Am J Health Promot. 2024;38:468–477. 10.1177/08901171231224864 38146733

[11] Taylor EV Brose LS McNeill A . Associations between smoking and vaping prevalence, product use characteristics, and mental health diagnoses in Great Britain: a population survey. BMC Med. 2023;21:211. 10.1186/s12916-023-02890-y PMC10268384 37316913

[12] Lindson N Butler AR McRobbie H, et al. Electronic cigarettes for smoking cessation. Cochrane Libr. 2025;1(1). CD010216. 10.1002/14651858.CD010216.pub9 PMC11776059 39878158

[13] Jackson SE Brown J Buss V . Prevalence of popular smoking cessation aids in England and associations with quit success. JAMA Netw Open. 2025;8:e2454962. 10.1001/jamanetworkopen.2024.54962 PMC11742533 39821398

[14] Jackson SE Brose L Buss V . Moderation of the real-world effectiveness of smoking cessation aids by mental health conditions: a population study. PLOS Ment Health. 2024;1:e0000007. 10.1371/journal.pmen.0000007 PMC12798440 41661824

[15] Zohara Z Abdul Jaffar Azad AR. Breaking the myths around E-Cigarettes: a narrative review exploring the impact of e-Cigarette on human health. Cureus. 2024;16:e72039. 10.7759/cureus.72039 PMC11578616 39569216

[16] Li Y Hecht SS. Carcinogenic components of tobacco and tobacco smoke: a 2022 update. Food Chem Toxicol. 2022;165:113179. 10.1016/j.fct.2022.113179 PMC9616535 35643228

[17] Blount BC Karwowski MP Shields PG . Vitamin E acetate in bronchoalveolar-lavage fluid associated with EVALI. N Engl J Med. 2020;382:697–705. 10.1056/NEJMoa1916433 PMC7032996 31860793

[18] Livingstone-Banks J Travis N Conde M . The impacts of e-cigarette flavours: an overview of systematic reviews. Addiction. 2025;120:1327–1344. 10.1111/add.70017 PMC12128567 39999998

[19] National Academies of Sciences, Engineering and Medicine. Public Health Consequences of E-Cigarettes. Washington, DC: The National Academies Press; 2018. Available at: https://nap.nationalacademies.org/catalog/24952/public-health-consequences-of-e-cigarettes. Accessed January 05, 2025.

[20] McNeill A Simonavičius E Brose L . Nicotine Vaping in England: An Evidence Update Including Health Risks and Perceptions. London: Office for Health Improvement and Disparities; 2022. Available at: https://www.gov.uk/government/publications/nicotine-vaping-in-england2022-evidence-update/nicotine-vaping-in-england-2022-evidence-update-summary#chapter-16-conclusions. Accessed January 05, 2025.

[21] Warner KE Benowitz NL McNeill A . Nicotine e-cigarettes as a tool for smoking cessation. Nat Med. 2023;29:520–524. 10.1038/s41591-022-02201-7 36788367

[22] National Institute for Health and Care Excellence. Tobacco: preventing uptake, promoting quitting and treating dependence; 2021. Available at: https://www.nice.org.uk/guidance/ng209/chapter/Recommendations-on-treating- tobacco-dependence#stop-smoking-interventions. Accessed January 1, 2025. 36745727

[23] United States Preventive Services Taskforce. Recommendation: tobacco smoking cessation in adults, including pregnant persons: interventions; 2025. Available at: https://www.uspreventiveservicestaskforce.org/uspstf/recommendation/tobacco -use-in-adults-and-pregnant-women-counseling-and-interventions. Accessed June 05, 2025

[24] Chavannes NH Meijer E Wind L . Herziene Richtlijn “Behandeling Van Tabaksverslaving En Stoppen Met Roken Ondersteuning: NTVG; 2025. Available at: https://www.ntvg.nl/artikelen/herziene-richtlijn-behandeling-van-tabaksverslaving-en-stoppen-met-roken-ondersteuning. Accessed June 05, 2025. 28401822

[25] NHS England. The NHS long term plan. [Internet]; 2019. Available at: https://www.longtermplan.nhs.uk/wp- content/uploads/2019/08/nhs-long-term-plan-version-1.2.pdf. Accessed January 17, 2025.

[26] Action on Smoking and Health (ASH). Space to breathe: findings from a survey of smokefree policies and tobacco dependence treatment services in NHS mental health trusts in England; 2024. Available at: https://ash.org.uk/resources/view/space-to-breathe-findings-from-asurvey-of-smokefree-policies-and-tobacco-dependence-treatment-services-innhs-mental-health-trusts-in-england-2024. Accessed January 05, 2025.

[27] NICE Guidance. Psychosis and schizophrenia in adults: prevention and management; 2014. Available at: https://www.nice.org.uk/guidance/cg178/chapter/Recommendations#promoting-recovery-and-possible-future-care. Accessed May 09, 2025.

[28] Taylor DM Barnes TRE Young AH. The maudsley prescribing guidelines in psychiatry (Full-Text access). In: Taylor DM Barnes TRE Young AH, eds, The Maudsley Prescribing Guidelines in Psychiatry; 2025. Available at: 10.1002/9781119870203.mpg016. Accessed January 17, 2025.

[29] NICE Clinical Knowledge Summaries, National Institute for Health and Care Excellence. Drugs Affected by Smoking Cessation (Revised Aug 2024); 2024. Available at: https://cks.nice.org.uk/topics/smoking-cessation/prescribinginformation/drugs-affected-by-smoking-cessation/. Accessed January 17, 2025.

[30] Clozapine Plus Werkgroep (Clozapine Plus Working Group). Richtlijn voor het gebruik van Clozapine (Guideline for the Use of Clozapine). Available at: https://www.clozapinepluswerkgroep.nl/publicaties/richtlijn-voor-het-gebruik-van-clozapine/. Accessed January 17, 2025.

[31] Grover S Mehta R Sah S . Expanding insights on clozapine dosing: the role of gender, smoking, and the clozapine/norclozapine ratio. J Clin Psychopharmacol. 2025;45:171–172. 10.1097/JCP.0000000000001954 39804775

[32] Harry NM Folorunsho IL Anona K . A review of the effect of vaping on the plasma levels of clozapine and its clinical implications. J Adv Med Med Res. 2024;36:48–56.

[33] Page MJ McKenzie JE Bossuyt PM . The PRISMA 2020 statement: an updated guideline for reporting systematic reviews. BMJ. 2021;372:n71. 10.1136/bmj.n71 PMC8005924 33782057

[34] Aromataris E Lockwood C Porritt K . JBI Manual for Evidence Synthesis: JBI; 2024. Available at: https://synthesismanual.jbi.global. Accessed January 17, 2025.

[35] Gagnier JJ Kienle G Alman DG . The CARE guidelines: consensus-based clinical case reporting guideline development. BMJ Case Rep. 2013;2:38–43. 10.7453/gahmj.2013.008 PMC3833570 24416692

[36] Nonner TSE Timmer SJ. Clozapine and the electronic cigarette; a case study. Tijdschr Psychiatr. 2016;58:314–317. 27075224

[37] Berm EJJL Ruijsbroek R Loonen AJM . Switching to E-cigarettes affects drug concentration. Ned Tijdschr Geneeskd. 2015;159:A9090. 26200424

[38] Khorassani F Kaufman M Lopez LV. Supatherapeutic plasma clozapine concentration after transition from traditional to electronic cigarettes. J Clin Psychopharmacol. 2018;38:391–392. 10.1097/JCP.0000000000000899 29901566

[39] Kocar T Freudenmann RW Spitzer M . Switching from tobacco smoking to electronic cigarettes and the impact on clozapine levels. J Clin Psychopharmacol. 2018;38:528–529. 10.1097/JCP.0000000000000948 30106884

[40] Montville DJ Lindsey JM Leung JG. Fluctuation between cigarette smoking and use of electronic nicotine delivery systems: impact on clozapine concentrations and clinical effect. Ment Health Clin. 2021;11:365–368. 10.9740/mhc.2021.11.365 PMC8582765 34824961

[41] Schoretsanitis G Haen E Conca A . Lack of smoking effects on pharmacokinetics of oral paliperidone-analysis of a naturalistic therapeutic drug monitoring sample. Pharmacopsychiatry. 2021;54:31–35. 10.1055/a-1221-5293 32767297

[42] Blacker CJ. Clinical issues to consider for clozapine patients who vape: a case illustration. Focus. 2020;18:55–57. 10.1176/appi.focus.20190025 PMC7011223 32047400

[43] Lam YWF. Altered clozapine concentrations associated with e-cigarettes. Brown Univ Psychopharmacol. 2018;30:2.

[44] de Leon J Schoretsanitis G Smith RL . An international guideline for making clozapine titration safer by using six ancestry based personalized dosing titrations, CRP, clozapine levels. Pharmacopsychiatry. 2021;55:73–86. 10.1055/a-1625-6388 34911124

[45] Wagner E McMahon L Falkai P . Impact of smoking behavior on clozapine blood levels - a systematic review and meta-analysis. Acta Psychiatr Scand. 2020;142:456–466. 10.1111/acps.13228 32869278

[46] Meyer JM. Individual changes in clozapine levels after smoking cessation: results and a predictive model. J Clin Psychopharmacol. 2001;21:569–574. 10.1097/00004714-200112000-00005 11763003

[47] Tsukahara M So R Yada Y . Changes in plasma clozapine levels after smoking cessation in Japanese inpatients with schizophrenia: a retrospective cohort study. Acta Med Okayama. 2022;76:137–143. 10.18926/AMO/63407 35503441

[48] Cormac I Brown A Creasey S . A retrospective evaluation of the impact of total smoking cessation on psychiatric inpatients taking clozapine. Acta Psychiatrica Scand. 2010;121:393–397. 10.1111/j.1600-0447.2009.01482.x 19824991

[49] Flanagan RJ Obee SJ Kim AHM . Plasma clozapine and N- desmethylclozapine (norclozapine) concentrations and the clozapine/norclozapine ratio. J Clin Psychopharmacol. 2024;44:492–501. 10.1097/JCP.0000000000001909 39173038

[50] Bersani FS Capra E Minichino A . Factors affecting interindividual differences in clozapine response: a review and case report. Hum Psychopharmacol. 2011;26:177–187. 10.1002/hup.1191 21455971

[51] Hukkanen J Jacob P III Peng M . Effect of nicotine on cytochrome P450 1A2 activity. Br J Clin Pharmacol. 2011;72:836–838. 10.1111/j.1365-2125.2011.04023.x PMC3243019 21599724

[52] McCarthy RH. Seizures following smoking cessation in a clozapine responder. Pharmacopsychiatry. 1994;27:210–211. 10.1055/s-2007-1014307 7838893

[53] Rostami-Hodjegan A Amin AM Spencer EP . Influence of dose, cigarette smoking, age, sex, and metabolic activity on plasma clozapine concentrations: a predictive model and nomograms to aid clozapine dose adjustment and to assess compliance in individual patients. J Clin Psychopharmacol. 2004;24:70–78. 10.1097/01.jcp.0000106221.36344.4d 14709950

[54] Lozano R Bona C. Influence of cigarette smoking habit on clozapine-to- norclozapine ratio in male patients. J Res Pharm Pract. 2023;12:29–31. 10.4103/jrpp.jrpp_37_23 PMC10779687 38213602

[55] Son Y Wackowski O Weisel C . Evaluation of E-Vapor nicotine and nicotyrine concentrations under various E-Liquid compositions, device settings, and vaping topographies. Chem Res Toxicol. 2018;31:861–868. 10.1021/acs.chemrestox.8b00063 PMC6350771 30080399

