# Supplementary material for: Effects of Switching From Smoking to Vaping on Clozapine Concentration–Dose Ratios and Plasma Clozapine Concentrations: A Systematic Review of Case Reports
Source: Ther Drug Monit. 2026 May 27;48(6):742–50. doi: 10.1097/FTD.0000000000001476 (PMC13747292; doi:10.1097/FTD.0000000000001476)
Supplement: Supplementary file 1 [file tdm-48-742-s001.docx]

Supplementary file 1- Detailed Search Strategy for all Databases.

# Cochrane.

#1	"electronic cigarette" or "electronic cigarettes" or e-cigarette or e-cigarettes or "e cigarette" or "e-cigarettes" or "electronic nicotine delivery systems" or "electronic nicotine delivery system" or "alternative nicotine delivery systems" or "alternative nicotine delivery system" or "personal vapouriser" or "personal vapourizer" or "personal vaporiser" or vaping or vapers:ti,ab,kw AND clozapine or olanzapine or antipsychotic* or anti-psychotic or anti- psychotics:ti,ab,kw (Word variations have been searched)

# Pubmed

((Electronic cigarette[Title/Abstract]) OR (E-cigarette[Title/Abstract]) OR (Electronic cigarettes[Title/Abstract]) OR (E-cigarettes[Title/Abstract]) OR (Electronic Nicotine Delivery System[Title/Abstract]) OR (Electronic Nicotine Delivery Systems[Title/Abstract]) OR (Alternative Nicotine Delivery System[Title/Abstract]) OR (Alternative Nicotine Delivery Systems[Title/Abstract]) OR (personal vaporizer[Title/Abstract]) OR (Vaping[Title/Abstract]) OR (Vapers[Title/Abstract) OR (Vapes[Title/Abstract)) AND ((Clozapine[Title/Abstract]) OR (antipsychotic*[Title/Abstract]) OR (anti- psychotic*[Title/Abstract]) OR (neuroleptic*[Title/Abstract]) OR (blood cell[Title/Abstract]) OR (agranulocytosis[Title/Abstract]) OR (CYP1A2[Title/Abstract]) OR (CYP 1A2[Title/Abstract]) OR (neutrophil*[Title/Abstract]) OR (Lymphocytes [Title/Abstract])) NOT EVALI

# CINAHL

TX ( Clozapine or antipsychotic* or anti-psychotic* or neuroleptic* or “blood cell” or agranulocytosis or CYP1A2 or “CYP 1A2” or neutrophil* or Lymphocytes ) AND TX ( “electronic cigarettes" or “electronic cigarette" or "e cigarettes" or e cigarette" or "e- cigarettes" or "e-cigarette" or "electronic vaping device*" or "electronic nicotine delivery systems" or “Electronic Nicotine Delivery System” or “Alternative Nicotine Delivery Systems” or “Alternative Nicotine Delivery System” or “personal vaporizer” or "vaping devices” or Vaping or vapers or vapes or E-cig* or “E cig*” ) NOT TX EVALI

**WoS**.

(ALL=("Electronic cigarettes") OR ALL=(E-cigarette) OR ALL=("Electronic cigarette") OR ALL=(E-cigarettes) OR ALL=("Electronic Nicotine Delivery System") OR ALL=("Electronic Nicotine Delivery Systems") OR ALL=("Alternative Nicotine Delivery System") OR ALL=("Alternative Nicotine Delivery Systems") OR ALL=("personal vaporizer") OR ALL=(Vaping) OR ALL=(Vapers) OR ALL=(Vapes) OR ALL=("E-cig*") OR ALL=("E cig*")) AND (ALL=(Clozapine) OR ALL=(antipsychotic*) OR ALL=(anti- psychotic*) OR ALL=(neuroleptic*) OR ALL=("blood cell") OR ALL=(agranulocytosis) OR ALL=(CYP1A2) OR ALL=("CYP 1A2") OR ALL=(neutrophil*) OR ALL=(Lymphocytes)) NOT (ALL=(EVALI ))

# OVID Medline and OVID Embase

| 1  . | "Electronic cigarette".mp. [mp=title, book title, abstract, original title, name of substance word, subject heading word, floating sub-heading word, keyword heading word, organism supplementary concept word, protocol supplementary concept word, rare disease supplementary concept word, unique identifier, synonyms, population supplementary  concept word, anatomy supplementary concept word] |
| --- | --- |
| 2  . | "Electronic cigarettes".mp. [mp=title, book title, abstract, original title, name of substance word, subject heading word, floating sub-heading word, keyword heading word, organism supplementary concept word, protocol supplementary concept word, rare disease supplementary concept word, unique identifier, synonyms, population supplementary  concept word, anatomy supplementary concept word] |
| 3  . | E-cigarette.mp. [mp=title, book title, abstract, original title, name of substance word, subject heading word, floating sub-heading word, keyword heading word, organism supplementary concept word, protocol supplementary concept word, rare disease supplementary concept word, unique identifier, synonyms, population supplementary concept word, anatomy  supplementary concept word] |
| 4  . | E-cigarettes.mp. [mp=title, book title, abstract, original title, name of substance word, subject heading word, floating sub-heading word, keyword heading word, organism supplementary concept word, protocol supplementary concept word, rare disease supplementary concept word, unique identifier, synonyms, population supplementary  concept word, anatomy supplementary concept word] |
| 5  . | "Electronic Nicotine Delivery System".mp. [mp=title, book title, abstract, original title, name of substance word, subject heading word, floating sub-heading word, keyword heading word, organism supplementary concept word, protocol supplementary concept word, rare disease supplementary concept word, unique identifier, synonyms, population  supplementary concept word, anatomy supplementary concept word] |
| 6  . | "Electronic Nicotine Delivery Systems".mp. [mp=title, book title, abstract, original title, name of substance word, subject heading word, floating sub-heading word, keyword heading word, organism supplementary concept word, protocol supplementary concept word, rare disease supplementary concept word, unique identifier, synonyms, population  supplementary concept word, anatomy supplementary concept word] |
| 7  . | "Alternative Nicotine Delivery System".mp. [mp=title, book title, abstract, original title, name of substance word, subject heading word, floating sub-heading word, keyword heading word, organism supplementary concept word, protocol supplementary concept word, rare disease supplementary concept word, unique identifier, synonyms, population  supplementary concept word, anatomy supplementary concept word] |
| 8  . | "Alternative Nicotine Delivery Systems".mp. [mp=title, book title, abstract, original title, name of substance word, subject heading word, floating sub-heading word, keyword  heading word, organism supplementary concept word, protocol supplementary concept |

|  | word, rare disease supplementary concept word, unique identifier, synonyms, population  supplementary concept word, anatomy supplementary concept word] |
| --- | --- |
| 9  . | "personal vaporizer".mp. [mp=title, book title, abstract, original title, name of substance word, subject heading word, floating sub-heading word, keyword heading word, organism supplementary concept word, protocol supplementary concept word, rare disease supplementary concept word, unique identifier, synonyms, population supplementary  concept word, anatomy supplementary concept word] |
| 1  0  . | Vaping.mp. [mp=title, book title, abstract, original title, name of substance word, subject heading word, floating sub-heading word, keyword heading word, organism supplementary concept word, protocol supplementary concept word, rare disease supplementary concept word, unique identifier, synonyms, population supplementary concept word, anatomy  supplementary concept word] |
| 1  1  . | Vapers.mp. [mp=title, book title, abstract, original title, name of substance word, subject heading word, floating sub-heading word, keyword heading word, organism supplementary concept word, protocol supplementary concept word, rare disease supplementary concept word, unique identifier, synonyms, population supplementary concept word, anatomy  supplementary concept word] |
| 1  2  . | Vapes.mp. [mp=title, book title, abstract, original title, name of substance word, subject heading word, floating sub-heading word, keyword heading word, organism supplementary concept word, protocol supplementary concept word, rare disease supplementary concept word, unique identifier, synonyms, population supplementary concept word, anatomy  supplementary concept word] |
| 1  3  . | E-cig*.mp. [mp=title, book title, abstract, original title, name of substance word, subject heading word, floating sub-heading word, keyword heading word, organism supplementary concept word, protocol supplementary concept word, rare disease supplementary concept word, unique identifier, synonyms, population supplementary concept word, anatomy  supplementary concept word] |
| 1  4  . | "E cig*".mp. [mp=title, book title, abstract, original title, name of substance word, subject heading word, floating sub-heading word, keyword heading word, organism supplementary concept word, protocol supplementary concept word, rare disease supplementary concept word, unique identifier, synonyms, population supplementary concept word, anatomy  supplementary concept word] |
| 1  5  . | Clozapine.mp. [mp=title, book title, abstract, original title, name of substance word, subject heading word, floating sub-heading word, keyword heading word, organism supplementary concept word, protocol supplementary concept word, rare disease supplementary concept word, unique identifier, synonyms, population supplementary concept word, anatomy  supplementary concept word] |
| 1  6  . | antipsychotic*.mp. [mp=title, book title, abstract, original title, name of substance word, subject heading word, floating sub-heading word, keyword heading word, organism supplementary concept word, protocol supplementary concept word, rare disease supplementary concept word, unique identifier, synonyms, population supplementary  concept word, anatomy supplementary concept word] |
| 1  7  . | anti-psychotic*.mp. [mp=title, book title, abstract, original title, name of substance word, subject heading word, floating sub-heading word, keyword heading word, organism supplementary concept word, protocol supplementary concept word, rare disease supplementary concept word, unique identifier, synonyms, population supplementary  concept word, anatomy supplementary concept word] |
| 1  8  . | neuroleptic*.mp. [mp=title, book title, abstract, original title, name of substance word, subject heading word, floating sub-heading word, keyword heading word, organism supplementary concept word, protocol supplementary concept word, rare disease supplementary concept word, unique identifier, synonyms, population supplementary  concept word, anatomy supplementary concept word] |
| 1  9  . | 1 or 2 or 3 or 4 or 5 or 6 or 7 or 8 or 9 or 10 or 11 or 12 or 13 or 14 |
| 2  0  . | blood cell.mp. [mp=title, book title, abstract, original title, name of substance word, subject heading word, floating sub-heading word, keyword heading word, organism supplementary  concept word, protocol supplementary concept word, rare disease supplementary concept |

|  | word, unique identifier, synonyms, population supplementary concept word, anatomy  supplementary concept word] |
| --- | --- |
| 2  1  . | agranulocytosis.mp. [mp=title, book title, abstract, original title, name of substance word, subject heading word, floating sub-heading word, keyword heading word, organism supplementary concept word, protocol supplementary concept word, rare disease supplementary concept word, unique identifier, synonyms, population supplementary  concept word, anatomy supplementary concept word] |
| 2  2  . | CYP 1A2.mp. [mp=title, book title, abstract, original title, name of substance word, subject heading word, floating sub-heading word, keyword heading word, organism supplementary concept word, protocol supplementary concept word, rare disease supplementary concept word, unique identifier, synonyms, population supplementary concept word, anatomy  supplementary concept word] |
| 2  3  . | CYP1A2.mp. [mp=title, book title, abstract, original title, name of substance word, subject heading word, floating sub-heading word, keyword heading word, organism supplementary concept word, protocol supplementary concept word, rare disease supplementary concept word, unique identifier, synonyms, population supplementary concept word, anatomy  supplementary concept word] |
| 2  4  . | neutrophil$.mp. [mp=title, book title, abstract, original title, name of substance word, subject heading word, floating sub-heading word, keyword heading word, organism supplementary concept word, protocol supplementary concept word, rare disease supplementary concept word, unique identifier, synonyms, population supplementary  concept word, anatomy supplementary concept word] |
| 2  5  . | lymphocytes.mp. [mp=title, book title, abstract, original title, name of substance word, subject heading word, floating sub-heading word, keyword heading word, organism supplementary concept word, protocol supplementary concept word, rare disease supplementary concept word, unique identifier, synonyms, population supplementary  concept word, anatomy supplementary concept word] |
| 2  6  . | EVALI.mp. [mp=title, book title, abstract, original title, name of substance word, subject heading word, floating sub-heading word, keyword heading word, organism supplementary concept word, protocol supplementary concept word, rare disease supplementary concept word, unique identifier, synonyms, population supplementary concept word, anatomy  supplementary concept word] |
| 2  7  . | 15 or 16 or 17 or 18 or 20 or 21 or 22 or 23 or 24 or 25 |
| 2  8  . | 19 and 27 |
| 2  9  . | 28 not 26 |

**APA Psych Info**

01 "Electronic cigarette".mp. [mp=title, abstract, heading word	126

, table of contents, key concepts, original title, tests & m easures, mesh word]

02 "Electronic cigarettes".mp. [mp=title, abstract, heading wor	437 d, table of contents, key concepts, original title, tests &

measures, mesh word]

03 E-cigarette.mp. [mp=title, abstract, heading word, table of	352 contents, key concepts, original title, tests & measures, me

sh word]

04 E-cigarettes.mp. [mp=title, abstract, heading word, table of	350 contents, key concepts, original title, tests & measures, m

esh word]

05 "Electronic Nicotine Delivery System".mp. [mp=title, abstrac	11 t, heading word, table of contents, key concepts, original t

itle, tests & measures, mesh word]

06 "Electronic Nicotine Delivery Systems".mp. [mp=title, abstra	151 ct, heading word, table of contents, key concepts, original

title, tests & measures, mesh word]

07 "Alternative Nicotine Delivery System".mp. [mp=title, abstra	1 ct, heading word, table of contents, key concepts, original

title, tests & measures, mesh word]

08 "Alternative Nicotine Delivery Systems".mp. [mp=title, abstr	4 act, heading word, table of contents, key concepts, original

title, tests & measures, mesh word]

09 "personal vaporizer".mp. [mp=title, abstract, heading word,	1

table of contents, key concepts, original title, tests & mea sures, mesh word]

10 Vaping.mp. [mp=title, abstract, heading word, table of conte	184

nts, key concepts, original title, tests & measures, mesh wo rd]

11 Vapers.mp. [mp=title, abstract, heading word, table of conte	33

nts, key concepts, original title, tests & measures, mesh wo rd]

12 Vapes.mp. [mp=title, abstract, heading word, table of conten	9

ts, key concepts, original title, tests & measures, mesh wor d]

13 E-cig*.mp. [mp=title, abstract, heading word, table of conte	438

nts, key concepts, original title, tests & measures, mesh wo rd]

14 "E cig*".mp. [mp=title, abstract, heading word, table of con	438

tents, key concepts, original title, tests & measures, mesh word]

15 Clozapine.mp. [mp=title, abstract, heading word, table of co	320

ntents, key concepts, original title, tests & measures, mesh word]

16 antipsychotic*.mp. [mp=title, abstract, heading word, table	1759

of contents, key concepts, original title, tests & measures, mesh word]

17 anti-psychotic*.mp. [mp=title, abstract, heading word, table	29

of contents, key concepts, original title, tests & measures

, mesh word]

18 neuroleptic*.mp. [mp=title, abstract, heading word, table of	867 contents, key concepts, original title, tests & measures, m

esh word]

19 1 or 2 or 3 or 4 or 5 or 6 or 7 or 8 or 9 or 10 or 11 or 12	565

or 13 or 14

20 blood [cell.mp](http://cell.mp/). [mp=title, abstract, heading word, table of c	66 ontents, key concepts, original title, tests & measures, mes

h word]

21 [agranulocytosis.mp](http://agranulocytosis.mp/). [mp=title, abstract, heading word, table	24 of contents, key concepts, original title, tests & measures

, mesh word]

22 CYP 1A2.mp. [mp=title, abstract, heading word, table of cont	1 ents, key concepts, original title, tests & measures, mesh w

ord]

23 CYP1A2.mp. [mp=title, abstract, heading word, table of conte	9 nts, key concepts, original title, tests & measures, mesh wo

rd]

24 neutrophil$.mp. [mp=title, abstract, heading word, table of	141 contents, key concepts, original title, tests & measures, me

sh word]

25 [lymphocytes.mp](http://lymphocytes.mp/). [mp=title, abstract, heading word, table of	152 contents, key concepts, original title, tests & measures, me

sh word]

26 EVALI.mp. [mp=title, abstract, heading word, table of conten	1 ts, key concepts, original title, tests & measures, mesh wor

d]

27 15 or 16 or 17 or 18 or 20 or 21 or 22 or 23 or 24 or 25	2259
28 19 and 27	2
29 28 not 26	2
30 limit 29 to updaterange="psyc17(20240515150811-2024052020472	0

0],psyc23(20240515150811-20240521152741]"
