# Supplementary material for: Effects of Switching From Smoking to Vaping on Clozapine Concentration–Dose Ratios and Plasma Clozapine Concentrations: A Systematic Review of Case Reports
Source: Ther Drug Monit. 2026 May 27;48(6):742–50. doi: 10.1097/FTD.0000000000001476 (PMC13747292; doi:10.1097/FTD.0000000000001476)
Supplement: Supplementary file 3 [file tdm-48-742-s003.docx]

Appendix 3- Completeness and Transparency of Case Reports using the CARE checklist^i^

| **First author year of publication** | **Title – The diagnosis or intervention of primary focus followed by the words “case report”.** | **Key Words – 2**  **to 5 key words that identify diagnoses or interventions in this case report (including "case report")** | **Abstract – (structured or unstructured d), *see more in reference below*** | **Introduction on – Briefly summarize es why this case is unique and may include medical literature references.** | **Patient Information** | **Clinical Findings – Describe significant physical examination (PE) and important clinical findings** | **Timeline**  **–**  **Historical and current information from this episode of care organized as a timeline (figure or table).** | **Diagnostic Assessment** | **Therapeutic Intervention on** | **Follow- up**  **&**  **Outcomes** | **Discussion** | **Patient Perspective** | **Inform ed Consent** |
| --- | --- | --- | --- | --- | --- | --- | --- | --- | --- | --- | --- | --- | --- |
| **Montville et al, 2021** | No | No | Yes | Yes | Yes | Yes | Yes | Yes | Yes | Yes | Yes | No | No  known |
| **Khorassani et al, 2018** | No | No | No | Yes | Yes | Yes | Yes | Yes | Yes | Yes | Yes | No | Yes |
| **Kocar et**  **al, 2018** | No | No | No | Yes | Yes | Yes | Yes | Yes | Yes | Yes | Yes | No | Yes |
| **Berm et**  **al, 2015** | No | No | Yes | Yes | Yes | Yes | Yes | Yes | Yes | Yes | Yes | No | Not  known |
| **Nonner et al,**  **2016** | No | No | No | Yes | Yes | Yes | Yes | Yes | Yes | Unclear | Yes | No | Not known |

i Gagnier JJ, Kienle G, Alman DG, Moher D, Sox H and Riley D. The CARE guidelines: consensus-based clinical case reporting guideline development. BMJ Case Rep. 2013; 2(5): 38-43. doi: https://doi.org/10.1136/bcr- 2013-201554.
